# COVID-19 Vaccine–Related Information on the WeChat Public Platform: Topic Modeling and Content Analysis

**DOI:** 10.2196/45051

**Published:** 2023-04-14

**Authors:** Xiaoqian Wu, Ziyu Li, Lin Xu, Pengfei Li, Ming Liu, Cheng Huang

**Affiliations:** 1 College of Medical Informatics Chongqing Medical University Chongqing China; 2 Department of Information Xiaoqiao Hospital Army Medical University (Third Military Medical University) Chongqing China; 3 School of Public Health Weifang Medical University Weifang China

**Keywords:** health belief model, COVID-19 vaccines, WeChat, content analysis, topic modeling, public health, COVID-19

## Abstract

**Background:**

The COVID-19 vaccine is an effective tool in the fight against the COVID-19 outbreak. As the main channel of information dissemination in the context of the epidemic, social media influences public trust and acceptance of the vaccine. The rational application of health behavior theory is a guarantee of effective public health information dissemination. However, little is known about the application of health behavior theory in web-based COVID-19 vaccine messages, especially from Chinese social media posts.

**Objective:**

This study aimed to understand the main topics and communication characteristics of hot papers related to COVID-19 vaccine on the WeChat platform and assess the health behavior theory application with the aid of health belief model (HBM).

**Methods:**

A systematic search was conducted on the Chinese social media platform WeChat to identify COVID-19 vaccine–related papers. A coding scheme was established based on the HBM, and the sample was managed and coded using NVivo 12 (QSR International) to assess the application of health behavior theory. The main topics of the papers were extracted through the Latent Dirichlet Allocation algorithm. Finally, temporal analysis was used to explore trends in the evolution of themes and health belief structures in the papers.

**Results:**

A total of 757 papers were analyzed. Almost all (671/757, 89%) of the papers did not have an original logo. By topic modeling, 5 topics were identified, which were vaccine development and effectiveness (267/757, 35%), disease infection and protection (197/757, 26%), vaccine safety and adverse reactions (52/757, 7%), vaccine access (136/757, 18%), and vaccination science popularization (105/757, 14%). All papers identified at least one structure in the extended HBM, but only 29 papers included all of the structures. Descriptions of solutions to obstacles (585/757, 77%) and benefit (468/757, 62%) were the most emphasized components in all samples. Relatively few elements of susceptibility (208/757, 27%) and the least were descriptions of severity (135/757, 18%). Heat map visualization revealed the change in health belief structure before and after vaccine entry into the market.

**Conclusions:**

To the best of our knowledge, this is the first study to assess the structural expression of health beliefs in information related to the COVID-19 vaccine on the WeChat public platform based on an HBM. The study also identified topics and communication characteristics before and after the market entry of vaccines. Our findings can inform customized education and communication strategies to promote vaccination not only in this pandemic but also in future pandemics.

## Introduction

The COVID-19 outbreak at the end of 2019 damaged societies and economies worldwide, endangering public health and leading to an unprecedented global public health and economic crisis. The World Health Organization (WHO) deemed it as a public health emergency of international concern by January 2020, and the outbreak was declared a pandemic in March [1,2]. The COVID-19 vaccine is one of the best ways to restore social function as an effective tool to combat the COVID-19 epidemic. The key to establishing herd immunity and preventing the spread of pandemics is by improving the speed and rate of vaccination. However, listed as one of the 10 threats to global health in 2019, vaccine hesitancy remains dire and growing globally [3]. Public hesitancy and reluctance to receive the COVID-19 vaccine impede the speed and coverage of vaccination and are major potential barriers to effective vaccine rollout [4]. Therefore, there is an urgent need to develop health education strategies that promote public trust and vaccine acceptance before and after vaccines are introduced to the market [5-7].

Web-based vaccine information dissemination is an important way to raise public awareness of vaccines. Multiple studies have shown that web-based vaccine information or social media interventions can influence audience vaccine behavior [8-11]. At the same time, with the rapid development of mobile internet and the popularity of social networks, social media has become the primary choice for the public to obtain various information. During the COVID-19 epidemic, social media became the main channel for disseminating health knowledge, official authoritative news, and disinformation due to its universality and convenience [9,12]. Culture may play a significant role in how vaccine is promoted based on health behavioral theory. Vaccine information on social media from different cultures and national contexts might differ, so it is necessary to analyze the spread of COVID-19 vaccine messages on Chinese social media. WeChat is the most widely and frequently used social media in China [13]. WeChat continued to occupy a pivotal position in the Chinese market after the COVID-19 outbreak. A study on public awareness and information dissemination of novel coronavirus pneumonia jointly conducted by the National Information Center and Nanjing University reported that WeChat is the most important channel for the public to obtain information on the outbreak [14]. Meanwhile, a report published by CSM Media Research reported that WeChat was the media with the longest user contact time during the time of the outbreak [15]. However, some characteristics of the COVID-19 vaccine–related papers widely circulated on WeChat remain unclear, such as the topics of interest to audiences at different stages and the source of the information in the papers. Therefore, we chose papers that caused widespread communication on WeChat public platforms as the research object and analyzed the topics and dissemination characteristics of the papers to help understand the types of information that trigger public attention.

The content of the information disseminated by the media is also crucial according to Social Cognitive Theory. Misinformation or inappropriate dissemination of information may have a significant impact on the acceptance of COVID-19 vaccine [16]. Information about infectious diseases and vaccines in social media may influence people’s health risks perception and their decision-making processes and risk management behaviors [17]. Therefore, it is critical to understand how social media promote the COVID-19 vaccine on the internet. However, there are still few studies evaluating papers related to COVID-19 vaccine from the WeChat platform. The rational application of health behavior theory could guarantee public health information dissemination effectively [18,19]. The health belief model (HBM) is one of the most widely used behavior change models [20]. It has proven its utility in explaining and predicting preventive health behaviors [21,22]. Moreover, HBM has been widely used to study the factors influencing vaccination intentions [23,24]. The structural variables it contains all showed utility in predicting influences on vaccination intention and uptake [25-27]. Previous studies examining the application of the human papillomavirus vaccine and Zika virus–related tweets to health behavior theory using the HBM [19,23]. One study examined the HBM expression in COVID-19 vaccine–related stories from 3 news outlets, but no study has introduced HBM to specifically assess the COVID-19 vaccine papers in Chinese social media [28]. Other than that, the expression of health belief structures in different topics remains unclear.

Therefore, this study aimed to systematically search for COVID-19 vaccine–related hot papers that trigger widespread dissemination on WeChat in order to identify the papers’ hot topics and dissemination characteristics. Based on HBM, this study evaluates the application of health behavior theory to COVID-19 pneumonia vaccine–related papers in WeChat and summarizes the reasons why the messages achieved a communication advantage. Explore the theory-driven COVID-19 vaccine information dissemination strategy, in order to more effectively persuade residents to vaccine not only during this pandemic but also during the future pandemic.

## Methods

### Data Collection

In September 2021, the total number of vaccinations in China exceeded 2.1 billion doses, and the proportion of the population completing the full course of vaccination met the herd immunization requirement [29,30]. On October 1, 2021, the daily increment of vaccination doses fell below 1 million doses for the first time until it recovered to more than 2 million doses on the 18th. Meanwhile, in mid-October, China carried out booster dose vaccinations one after another nationwide, and the first round of nationwide vaccinations was completed. This study focuses on the characteristics of the paper that caused widespread dissemination around the time the COVID-19 vaccine entered the market. In WeChat, papers with more than 100,000 reads are marked as “100000+,” which is considered a sign of widespread communication [31]. Therefore, we use the keyword “COVID-19 Vaccine” on the search engine of Qingbo big data platform to collect WeChat papers with more than 100,000 hits on October 18, 2021, as the search deadline. Qingbo big data is China’s largest third-party new media big data evaluation and research platform serving the Chinese government, China’s top news media, and internet companies including Tencent [32]. The organization covers the data of almost all major Chinese social media platforms, including WeChat, and is the largest third-party WeChat database in China [33,34]. It enjoys a high reputation among researchers and decision makers of China’s new media impact assessment standards and is often used as a data source by researchers [33,35].

Two independent researchers reviewed each potentially suitable paper. Inclusion criteria are that papers must be related to COVID-19 vaccines and written in Chinese. The following papers were excluded because (1) they focused on content unrelated to health communication, such as praising volunteers or scientists involved in vaccine development or promotion and financial news related to the COVID-19 vaccine, (2) they were deleted by the publisher and cannot be accessed, (3) they were presented in video form without substantive text, and (4) the content was duplicative (if the paper is a duplicate, we will keep the original paper, and if the original paper is not indicated, we will keep the paper with the highest number of likes).

### Content Analysis

The general features of the papers are recorded, including paper push location, content presentation, pushing time, original logo, information source, and publish subject. A coding protocol for content analysis is developed, tested, and implemented for the coding process. According to the HBM, the likelihood of people adopting a particular health-promoting behavior is influenced by the following variables: (1) perceived susceptibility to subjective perception of the possibility of health risks, (2) perceived severity of the consequences of contracting the disease, (3) perceived benefits of adopting a particular health behavior, (4) perceived barriers to the adoption of specific health behaviors, (5) self-efficacy of an individual’s assessment of confidence in being able to successfully perform a health behavior, and (6) behavioral cues that guide individuals to perform healthy behaviors [19,36]. Based on the original definition of the HBM variables, we redefined the variables of the HBM according to the information dissemination characteristics of the COVID-19 vaccine–related domain, as shown in Table 1. The role of health care professionals and celebrities in vaccine promotion has been emphasized several times in previous studies [37-41]. Trust in the government and vaccination coverage, at the same time, both influence the public’s vaccination willingness [38,40,42]. Therefore, we added social preference and defined social preference as an individual’s, government’s, or society’s attitude toward COVID-19 vaccine.

**Table 1:** Coding variables and descriptions based on health belief model.

Assessment variables	Definition and descriptions
Susceptibility description	The description of COVID-19 susceptibility or risk of possible infection in the paper
Severity description	The description of the consequences or threats to individuals of SARS-CoV-2 virus infection or refusal of vaccination
Benefits description	The description of the positive effects or benefits of vaccination in the paper
Solutions to obstacles	The description of the answer and solution to potential barriers or difficulties in an individual’s vaccination behavior
Self-efficacy and behavioral cues	The description of the conditions associated with the individual performing the act of vaccination or the ease of access to vaccines
Social preference	The description of the attitude of individuals, government, or society toward vaccination behavior

NVivo 12 (QSR International) was used to manage and code the samples. A single paper is the sample, independent natural sentence is the unit of analysis, and the semantic connotations of the sentences are the basis for coding. If sentence contains multiple connotations, it will be coded into multiple categories. If the same connotation is repeatedly expressed by consecutive sentences, they are combined and coded into the same category. Prior to coding, the 2 coders were trained in content analysis, coding guidelines, and NVivo 12 software operation until they reached a high level of agreement on understanding and using the coding protocol. More than 10% (100/757, 13%) of the identified hot papers were randomly selected for double coding, and reliability tests between content analysis codes were performed using κ coefficients. The first coder coded the remaining papers once the reliability was reached. Samples with differences in the reliability check were solved through discussion until the researchers agreed upon the results and were included in the results after recoding and approval.

### Topic Model Training

Prior to the topic modeling, we preprocessed the text data to avoid the noise that affects the topic modeling. The *jieba* package in Python programming environment was used to conduct word segmentation. Several common Chinese deactivation word lists, such as the Baidu deactivation word list, were used as the base word list. Based on this, high-frequency but nonactual words were added according to the content of the study. After deduplication and integration, our own deactivation word list was built. Domain-specific research requires the construction of relevant domain lexicons to improve the effect of word segmentation. We built a user lexicon based on the initial word segmentation results, combined with specific expressions related to the COVID-19 vaccine, to avoid some fixed collocations being further sliced and diced.

**Figure 1:**
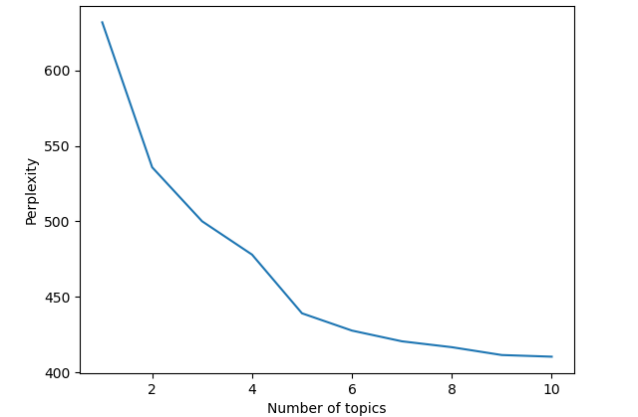
The topic perplexity score curve used to evaluate the performance of the topic model.

We used the Latent Dirichlet Allocation (LDA) algorithm for topic modeling. As a common and mature topic analysis method, LDA topic model is a Bayesian model including words, topics, and documents in 3 levels. The basic idea is that a document is composed of different potential topics according to a certain probability distribution, and each topic is composed of different words according to a certain probability distribution. The number of topics is an important parameter of the LDA and needs to be set manually. To determine the optimal number of topics with favorable model performance, we use the perplexity. Perplexity is a common method of determining the number of topics, with lower perplexity showing better model fit [43]. Increasing the number of topics usually decreases the perplexity, but too large a number of topics can cause the model to overfit and lead to difficulties in interpretation and subjective validation. As shown in Figure 1, the perplexity decreases as the number of topics increases. When the number of topics is 5, there is an obvious inflection point of the perplexity curve, that is, the curve decreases sharply when the number of topics is less than 5, and the curve basically tends to be smooth when the number of topics is greater than 5. The selection of the number of topics relying only on the application of statistical methods is often difficult to interpret for humans [44]. Therefore, we further evaluated the model effect by varying the number of topics. Compared to other topic models, when the number of topics is 5, the topic model strikes a balance between being too narrow that may ignore important content and too broad that makes it difficult to interpret. Therefore, we chose 5 as our topic number.

### Ethical Considerations

This study was approved by the Ethics Committee of Chongqing Medical University.

## Results

### General Characteristics

**Figure 2:**
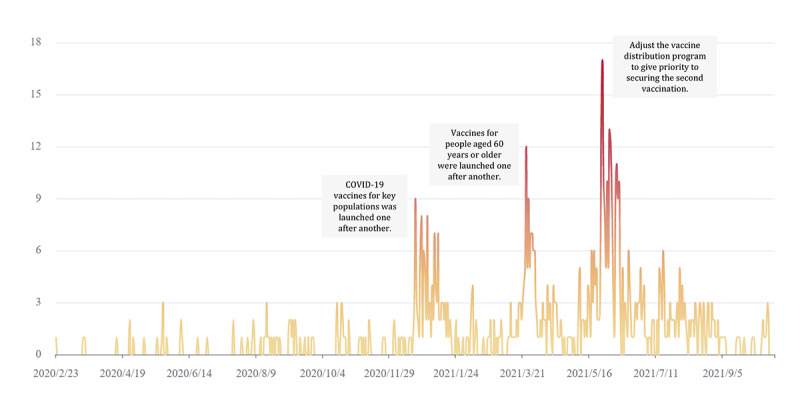
Daily number of papers with more than 100,000 reads related to COVID-19 vaccines from February 23, 2020, to October 18, 2021.

We identified a total of 757 papers. The number of papers is driven by the vaccine allocation program. As shown in Figure 2, the number of papers reached a stage peak when the government released a program related to vaccine distribution. For example, the number of papers with more than 100,000 reads peaked at 17 on May 27, 2021, when the vaccine distribution schedule was adjusted to prioritize the second dose. As shown in Table 2, most papers were published in headline format accounting for 79% (597/757) of the total. The main form of content is the combination of pictures and text (566/757, 75%). Original logos were lacking in almost all of the papers (671/757, 89%). More than half (454/757, 60%) of the papers marked the editors or journalists of the paper, which was the most predominant way. Overall, 423 papers marked that the message came from other WeChat official accounts. This was followed by government departments, with 211 papers labeled as having a government department as the information source. The Center of Disease Control (98/211, 46%) and the Health and Welfare Commission (96/211, 45%) were the most prominent government department sources.

**Table 2:** General characteristics collected for each of the papers.

Assessment measure			Papers, n (%)
**Paper push location**			
	Headlines	597 (79)	
	Subheadlines	160 (21)	
**Content presentation**			
	Pure text	98 (13)	
	Combination of text and picture	566 (75)	
	Combination of video, text, and picture	93 (12)	
**Original logo**			
	Yes	86 (11)	
	No	671 (89)	
**Information source**			
	Editors or journalists	454 (60)	
	Other WeChat official accounts	423 (56)	
	Government departments	211 (28)	
	Occupational physicians or medical practitioners	40 (5)	
	Expert researchers	44 (6)	
	Scientific research institution	7 (1)	
	Scientific literature	39 (5)	
	Medical institution	28 (4)	
	Not indicated	93 (12)	

### Health Belief Structure in the Paper

A total of 100 identified hot papers were randomly selected for double coding to check the reliability test between content analysis codes. The κ coefficient was 0.84, and greater than 0.8 was considered reliable. A total of 8929 reference points were recorded based on HBM with an average of 12 reference points per paper. As shown in Table 3, solutions to obstacles (585/757, 77%) were the most prominent category, mentioned more than 3 times per paper on average. The burden of vaccination procedures appears in most papers and contains requirements for matters in the vaccination process, such as vaccine injection requirements, interval requirements for immunization procedures, and the burden of vaccination matters. The second is vaccine side effects, which mainly include the incidence and severity of adverse reactions to vaccines and the way to deal with them. Immunization benefits were mentioned the most in the descriptions of vaccine benefits, 114 of which (114/354, 32%) gave specific effectiveness data. Social benefits were relatively less frequently mentioned, with 258 papers, more than half of which (164/258, 64%) emphasized the benefits of establishing an immune barrier to protect the population by achieving mass vaccination. Self-efficacy and behavioral cues descriptions depicted the conditions of access to vaccines, allowing people to assess their confidence in obtaining the vaccine. Expression of vaccine accessibility by describing the time and place of vaccination was the predominant way (379/455, 83%). About half (232/455, 51%) of the papers additionally mentioned vaccine allocation, which is the basis for equity in vaccine access, with vaccines being given priority to high-risk populations based on prevention and control considerations. Social preference emphasizes psychosocial factors and is a health belief variable increased according to the characteristics of the COVID-19 vaccine. Vaccination status by country or region appeared in the largest number of papers. The attitudes of authority figures (eg, health care professionals and presidents) had fewer papers than national or regional vaccination situation but were mentioned 1.21 times per paper, which was higher than the description of vaccination situation. Susceptibility and severity describe what may pose as a perceived threat to people. Susceptibility descriptions were mainly focused on descriptions of the epidemic situation (164/208, 79%), such as new cases in the country or region, the emergence of mutant strains, and changes in risk areas. Severity descriptions were mainly focused on descriptions of health threats, and descriptions of social consequences for life or work were only present in 35 papers.

**Table 3:** Results of a content analysis review based on the health belief model.

Variable			Number of papers		Reference points		Average number of mentions per paper
**Susceptibility description**			208		284		1.37
	The current situation of COVID-19 at home and abroad	164		190		1.16	
	The expression of novel coronavirus infection characteristics	78		94		1.21	
**Severity description**			135		173		1.28
	The threat to life and health after novel coronavirus infection	110		132		1.20	
	The impact of unvaccinated on life or work	35		41		1.17	
**Benefits description**			468		879		1.88
	The immune benefits	354		550		1.55	
	The benefits to society	258		329		1.28	
**Solutions to obstacles**			585		1874		3.20
	Economic burden	132		144		1.09	
	Side effects of vaccines	288		377		1.31	
	The burden of vaccination procedures	333		526		1.58	
	Safety of vaccines	273		373		1.37	
	Limitations of vaccines	121		147		1.21	
	Vaccination taboos and people delaying vaccination	249		307		1.23	
**Self-efficacy and behavioral cues**			455		825		1.81
	Vaccine accessibility	379		537		1.42	
	Vaccine allocation	232		288		1.24	
**Social preference**			360		567		1.58
	The attitude of authoritative personnel	123		149		1.21	
	Government policy support for vaccination	97		111		1.14	
	National or regional vaccination situation	205		236		1.15	
	International willingness to accept the vaccine	61		71		1.16	

### Topics Identified by the Topic Model

#### Overview

**Figure 3:**
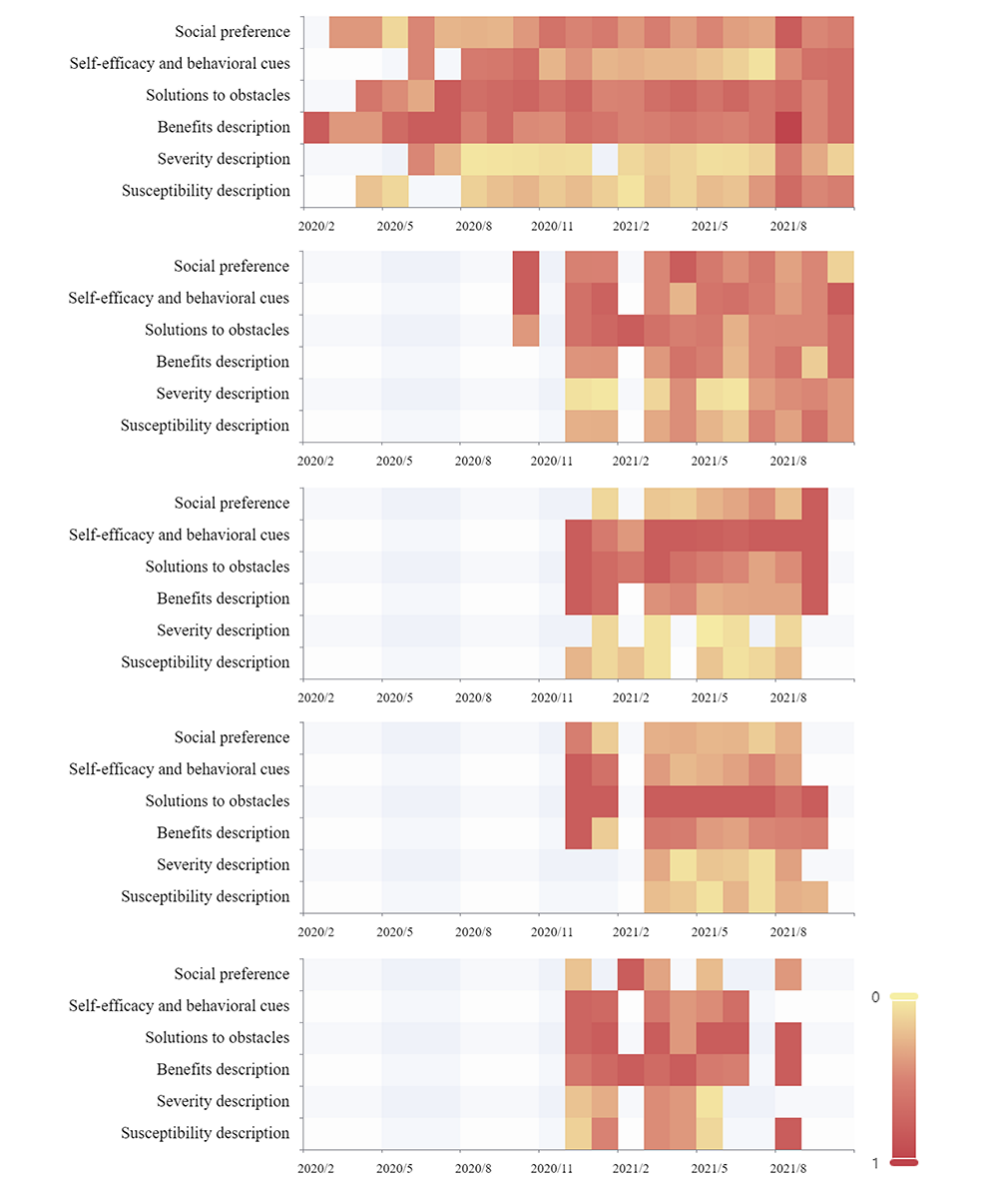
Heat map of the evolution of health belief constructs for each theme, from top to bottom, topic 1 (vaccine development and effectiveness), topic 2 (disease infection and protection), topic 3 (vaccine access), topic 4 (vaccination science popularization), and topic 5 (vaccine safety and adverse reactions). The x-axis represents the months during the study period. Darker colors correspond to higher prevalence of health belief structures (defined by the ratio of the specific construct–related papers to total HBM-related papers).

We identified 5 topics and their keywords and assigned the highest probability topic to each paper, as shown in Table 4. We further defined the prevalence of each HBM variable in each topic by calculating the ratio of the number of papers containing the structure to the total number of HBM-related papers (Figure 3).

**Table 4:** Topics and keywords formulated by Latent Dirichlet Allocation topic modeling.

Theme and topics	Keywords	Papers, n (%)
Vaccine development and effectiveness	Research, infection, clinical trial, antibody, data, protection, Sinopharm, CanSinoBIO, variant, clinical, trial, safety, Sinovac, effectiveness, effect, injection, prevention, institute	267 (35)
Disease infection and protection	Prevention and control, case, confirmed, pneumonia, risk, newly added, nucleic acid, input, detection, mask, protection, area, spread, cumulative, isolation	197 (26)
Vaccine access	Appointment, vaccination site, second dose, first dose, citizen, resident, community, registration, arrangement, public, service center, identity card, community health, queue	136 (18)
Vaccination science popularization	Disease, acute, drugs, component, allergy, interval, vaccination taboos, post-vaccination, deferred vaccination, diabetes, pre-vaccination, hypertension, fever, lactation, symptoms, pregnancy, chronic illness	105 (14)
Vaccine safety and adverse reactions	Post-vaccination, protection, monitoring, effect, antibody, safety, protein, adenovirus vector vaccine, injection site, recombinant, variant, redness, swelling, coupling, fever, pain	52 (7)

#### Topic 1: Vaccine Development and Effectiveness

**Figure 4:**
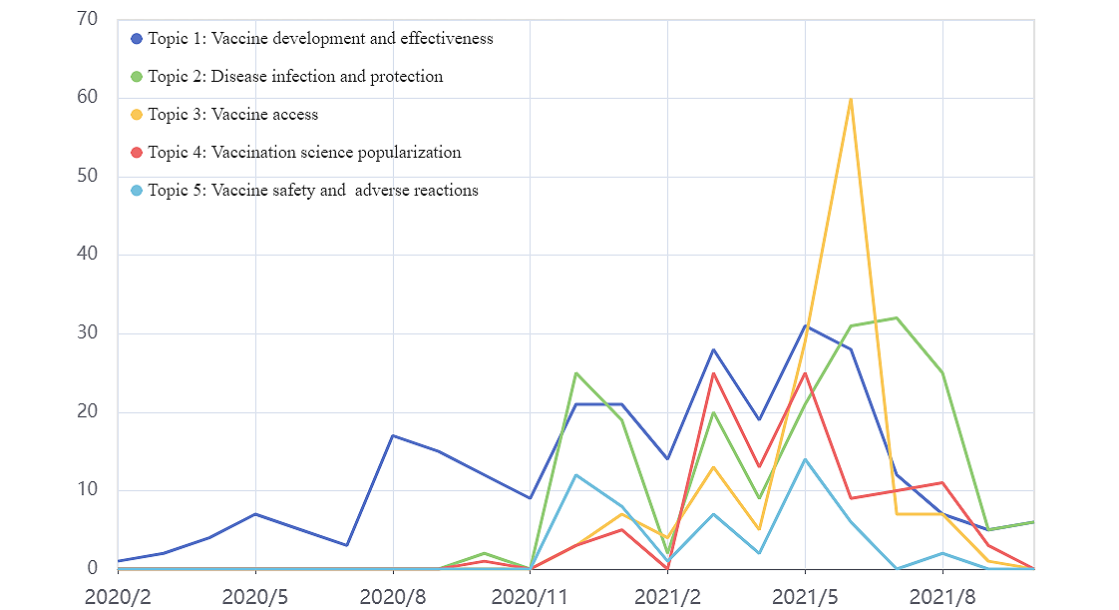
Monthly frequency of each topic on WeChat from February 2020 to October 2021.

Hot papers with vaccine development and effectiveness as the main theme run throughout the examination, peaking in May and June 2021 (Figure 4). This may be related to the announcement by the WHO in these 2 months to place vaccines from Sinopharm and Sinovac on the emergency use list. More than 2 in 3 papers under this theme described both benefits and solutions to obstacles (Table 5) and maintained a higher share than the other constructs throughout the study period. Benefits description was achieved mainly by emphasizing the immunizing effect of the vaccine, which was mentioned in 163 of 194 papers. Explanations of vaccine safety (122/210) and side effects (103/210) to alleviate public perceptions of barriers to vaccination were the most frequently mentioned elements. Severity and susceptibility were described relatively infrequently but were largely consistent throughout the study period.

**Table 5:** The performance of health belief structures in each topic.

Variable	Topic 1: vaccine development and effectiveness (N=267), n (%)	Topic 2: disease infection and protection (N=197), n (%)	Topic 3: vaccine access (N=136), n (%)	Topic 4: vaccination science popularization (N=105), n (%)	Topic 5: vaccine safety and adverse reactions (N=52), n (%)
Susceptibility description	71 (27)	81 (41)	19 (14)	21 (20)	16 (31)
Severity description	37 (14)	50 (25)	10 (7)	26 (25)	12 (23)
Benefits description	194 (73)	108 (55)	62 (46)	63 (60)	41 (79)
Solutions to obstacles	210 (79)	130 (66)	93 (68)	103 (98)	49 (94)
Self-efficacy and behavioral cues	104 (39)	139 (71)	127 (93)	48 (46)	37 (71)
Social preference	146 (55)	121 (61)	47 (35)	34 (32)	12 (23)

#### Topic 2: Disease Infection and Protection

The number of hot papers on this topic is closely related to the epidemic situation, for example, the number of hot papers peaked in July 2021 when imported cases from abroad and local cases appeared one after another in some parts of China, and public attention shifted to the disease itself. Each of the health belief constructs in this theme, except for the severity description, was occurring at a high frequency after December. The proportion of papers containing descriptions of severity increased month by month, eventually equalizing the prevalence of each health belief construct. The number of papers containing all 6 health belief constructs also reached 15, the highest percentage of all topics. In addition, the description of susceptibility had the highest proportion of all themes, with a predominance of descriptions of the domestic and international epidemic situation (70/81, 86%).

#### Topic 3: Vaccine Access

Self-efficacy and behavioral cues were the most prominent health belief construct in this topic, mentioned in almost all papers (127/136, 93%). Except for a small downward adjustment in early 2021, they all maintain the highest prevalence. Representing vaccine accessibility by accounting for information such as the time and place of vaccination is the most common way this construct is used in this topic (121/127). In December 2020, vaccination programs were announced and papers related to topic 3 (vaccine access) entered the public domain. As can be seen in Figure 3, there were more papers categorized in 3 constructs of self-efficacy and behavioral cues, benefits, and solutions to obstacles in the previous period. This may be related to the hesitant attitude of some members of the public toward vaccination in the early stages of vaccination. The public is concerned about the benefits of vaccination and the interpretation of barriers to vaccination, in addition to understanding how to obtain the vaccine. As the number of vaccinations increased, public sentiment toward vaccination gradually increased and the proportion of papers containing descriptions of benefits and barriers diminished. Monthly vaccination amount increased by more than 500 million doses in June 2021, reaching a recent high. The number of papers for topic 3 (vaccine access) also peaked this month (Figure 4).

#### Topic 4: Vaccination Science Popularization

In March 2021, the National Health Commission released the *COVID-19 Vaccination Guidelines (First Edition)*, and the hot papers with the main topic of vaccination science continued to attract public reading, and the number of papers continued to be second only to topic 1 for 2 months (Figure 4). Almost all of the papers (103/105, 98%) provide answers to possible barriers that the public may encounter in the vaccination process. By analyzing the prevalence of health belief constructs, we found that the barrier construct has remained at a stable and highest level since the announcement of the vaccination program in December 2020. The science of contraindications to vaccination for specific populations was the most frequently mentioned solutions to obstacles, which also corresponds to the keywords diabetes, hypertension, and pregnancy in the results of the topic modeling.

#### Topic 5: Vaccine Safety and Adverse Reactions

Topic 5 was used as the main theme less frequently than other topics for most of the period we examined. We note that topic 5 has 2 peaks in December 2020 and May 2021, respectively. By reviewing the papers from both time periods, the reasons that led to the interest in this topic were identified. A 2-step vaccination program was announced in December 2020, and the COVID-19 vaccine, which requires only one dose, was officially launched in May 2021, making the safety of the vaccine a hot topic of public concern. Similar to topic 4, almost all papers in topic 5 mentioned explanations for barriers to vaccination (49/52, 94%).

## Discussion

### Principal Findings

The media plays an important role in raising public awareness about preventive behaviors in the context of major outbreaks, so it is critical to understand the role of media in mobilizing the public for vaccination with the COVID-19 vaccine. Previous studies have documented how various variables of the HBM influence public vaccination behavior, but few studies have evaluated vaccine promotion papers in social media. This study is the first to borrow from HBM to comprehensively compare and evaluate popular papers related to the COVID-19 vaccine that have been widely disseminated on WeChat, a representative social media in China. We analyzed the trends in the evolution of COVID-19 vaccine–related paper topics and the prevalence of health belief structures within each topic before and after the market entry of the COVID-19 vaccine. The study can serve as a reference for implementers responsible for vaccine promotion as an important theoretical basis for responding to future vaccine promotion under public health emergencies. In an era of rapid internet globalization, this study may also provide a reference for other countries to use data from social media platforms (eg, Twitter and Facebook) to conduct relevant research and improve information governance during public health emergencies.

The release dates of relevant policies constitute the time points for the proliferation of paper innovations. The analysis of each theme also shows this pattern. The combination of graphics and text is the most prominent form of presentation in the hot papers, accounting for 75% (566/757) of the total. Therefore, interpretation and analysis of relevant policies and the use of appropriate multimedia may be a way to attract readers’ attention. Sources are an important indicator of the authenticity and credibility of a paper, and the addition of reliable sources to show credibility is necessary [45]. Trusted sources of information will be more effective, especially when health professionals are involved [46]. However, the sources of only 40 of the 757 papers we identified included occupational physicians or medical practitioners. Only 44 papers indicated the involvement of expert researchers, and the information came mainly from editors or journalists themselves. Journalists lack the relevant expertise and will have serious repercussions if they disseminate inappropriate or incorrect health information, which in turn will affect COVID-19 vaccine acceptance [16]. WHO also stresses the importance of using expert and knowledgeable resources or consulting with scientific media centers in each country when reporting on the COVID-19 vaccine [47].

Interventions with a clear theoretical basis are more effective compared with those that lack a clear theoretical basis [20]. The HBM was one of the first health behavior theories, which has been widely used in vaccination behavior research. Although all papers contain at least 1 construct, only 29 papers containing all 6 constructs are inadequate. Perceived barriers and perceived benefits are the strongest predictors of behavior in the HBM [48]. Corresponding to this study, these 2 constructs are also the most prominent. Safety, side effects, and efficacy were all present in less than half of the papers. However, vaccine-hesitant individuals were most concerned about vaccine efficacy and side effects in previous studies [38]. On the other hand, the COVID-19 vaccine’s development is more rapid than that of previous vaccines due to its specificity, and the impact of speed on safety and efficacy has become a matter of public concern. Answers on this are currently lacking. This all underscores the need for the media to step up their advocacy for the safety and efficacy of vaccines. Health workers are the most trusted source of COVID-19 vaccine guidance [49]. However, this part of the paper is also inadequately expressed, with only 123 papers (less than one-fifth) expressing the attitudes of authoritative personnel regarding vaccination. Emphasizing the efficacy and safety of vaccines through authority figures such as health workers may be an effective way to reduce vaccine hesitation.

Our findings suggest that both the number of papers and the structural prevalence of health beliefs in each topic have undergone substantial temporal changes, which may indicate the evolution of WeChat users’ hotspots of interest. After the announcement of the vaccination program in December 2020, the public attention did not change to vaccination science and vaccine access, which may be related to the continued lack of confidence in vaccines in the early stages of vaccine promotion. We note that the prevalence of the social preference structure in topic 3 was lower during this period relative to the other periods, while other topics have a high level in this period. Topic 3 (vaccine access) targeted the public who already held vaccination intentions. The high level of prevalence of social preference structure in other topics may reflect the concern of people who hesitated about vaccines on social preferences in the early stage of vaccine promotion. Indeed, positive social preferences such as influential figures’ attitudes toward vaccination and regional vaccination rates have been shown to increase public confidence, which in turn promotes vaccination [50]. Therefore, increasing social preference promotion of positive behavioral intentions in the early stages of vaccine promotion to create a positive social impact may be a viable approach.

The percentage of obstacles solution structures was high for all topics and did not decrease significantly in prevalence during this study time period. Reducing the public's assessment of the risk and benefit ratio of vaccination continues to be the focus of media campaigns. The prevalence of susceptibility and severity constructs across topics was not high during most of the study period, which may be related to the fact that both descriptions are based on fear-based appeals that may trigger public perceptions of threat. This may be related to the significant damage caused by this COVID-19 pneumonia outbreak, and the publisher’s fear that fear-based appeals will cause public anxiety and fear, which in turn will cause the public to avoid such information and preventive health behaviors [51]. This is consistent with previous studies in which public health agencies are generally reluctant to broadcast emotionally charged propaganda [51]. However, people are more inclined to content that is closely related to them in risk communication. Appropriate fear-based appeals are effective in influencing attitudes toward healthy behaviors and help to motivate the adoption of health behaviors [52]. At the same time, previous studies have disproved the publishers’ fears of lawsuits [52]. Combining the vaccination efficacy claims with the severity of susceptibility to COVID-19 is an approach worth considering. The susceptibility and severity of fear-based descriptions promote public vaccination behavior, while the efficacy of the vaccine alleviates public anxiety and fear.

### Limitations and Future Work

The study continues to have some limitations. We may have missed some papers as the keyword search cannot exhaust all papers. Although the search strategy can be extended to SARS-CoV-2 virus to identify more relevant papers, the search results are overwhelming for us to perform manual content analysis. On the other hand, due to censorship, some papers may be removed before data collection. However, this has minimal impact on our results because the WeChat public platform reviews the content of papers once before they are sent out, so only a very small percentage of papers will be deleted due to censorship after they are sent out. This study evaluated the use of the theoretical framework in the WeChat hot papers, but due to the limited expertise of the researchers, we did not assess the degree of authoritative science in the content of the papers. We will consider adding subsequent researchers to continue the study in-depth. Researchers could focus on web-based vaccine promotion papers in other languages in future researches. In addition, the readability and applicability of the papers also affect the user’s information acceptance, and future studies could further evaluate papers with the help of readability measures.

### Conclusions

This study describes the topics and communication characteristics of information related to the COVID-19 vaccine that was widely disseminated on WeChat public platforms before and after its introduction into the market. For the first time, we assessed the application of their health behavior theory by using an HBM. Additionally, we explored trends in the evolution of themes and trends in the prevalence of health belief structures across themes. This can help researchers identify time-based hotspots of public interest to design-targeted communication strategies to promote vaccination not only in this pandemic but also in future pandemics. Overall, the structure of the HBM included in the paper is inadequate. Barrier and benefit descriptions were performed most prominently, but the descriptions of safety and effectiveness, which are of concern, continue to need strengthening. The lack of trusted sources for the labeling of the paper may raise concerns about confidence in the authenticity of authority. The use of publicly trusted health workers to advocate for guidance on vaccination is worth considering.

## Abbreviations

HBM: health belief model
LDA: Latent Dirichlet Allocation
WHO: World Health Organization

## Data Availability

The data sets generated and analyzed during this study are available from the corresponding author on reasonable request.
